# ﻿Updated taxonomic keys for European Hippoboscidae (Diptera), and expansion in Central Europe of the bird louse fly *Ornithomyacomosa* (Austen, 1930) with the first record from Slovakia

**DOI:** 10.3897/zookeys.1115.80146

**Published:** 2022-07-29

**Authors:** Jozef Oboňa, Katarína Fogašová, Miroslav Fulín, Stanislav Greš, Peter Manko, Jakub Repaský, Jindřich Roháček, Oldřich Sychra, Martin Hromada

**Affiliations:** 1 Laboratory and Museum of Evolutionary Ecology, Department of Ecology, Faculty of Humanities and Natural Sciences, University of Prešov, 17. novembra 1, SK – 081 16 Prešov, Slovakia; 2 Puškinova 15, SK – 083 01 Sabinov, Slovakia; 3 17. novembra 24, SK – 083 01 Sabinov, Slovakia; 4 Ražňany 407, SK – 082 61 Ražňany, Slovakia; 5 Department of Entomology, Silesian Museum, Nádražní okruh 31, CZ – 746 01 Opava, Czech Republic; 6 Department of Biology and Wildlife Diseases, Faculty of Veterinary Hygiene and Ecology, University of Veterinary Sciences Brno, Palackého tř. 1946/1, CZ – 612 42 Brno, Czech Republic; 7 Faculty of Biological Sciences, University of Zielona Góra, PL – 65-417 ZielonaGóra, Poland

**Keywords:** Birds, hippoboscid, new host records, new record, parasite, Slovakia, taxonomic keys

## Abstract

The available keys for European Hippoboscidae are outdated and do not cover all species currently known from Europe. Therefore, identification keys to the eleven genera and 31 species of the European hippoboscids are provided here. *Ornithomyacomosa* (Austen, 1930) (Diptera: Hippoboscidae) is recorded for the first time from the territory of Slovakia based on one female found on a sand martin, *Ripariariparia* (Linnaeus, 1758). The list of keds and louse flies recorded from the territory of Slovakia is increased to 20 species. New host records for Slovakia are presented.

## ﻿Introduction

Keds and louse flies (Diptera: Hippoboscidae) are among the most fascinating as well as disregarded group of blood-feeding ectoparasites, and they thrive on many animal species (Bezerra-Santos and Otranto 2020). This family is included in the superfamily Hippoboscoidea, along with the families Glossinidae (tse-tse flies), Streblidae, and Nycteribiidae (bat flies) (Petersen et al. 2007; Reeves and Lloyd 2019). Hippoboscidae are divided into the subfamilies Lipopteninae (tribe Lipoptenini parasitising exclusively mammals), Ornithomyinae (tribes Olfersiini and Ornithomyini composed of species that mostly parasitise birds) and Hippoboscinae (tribe Hippoboscini with all species in Europe affecting mammals) (Reeves and Lloyd 2019). Phylogenetic studies have indicated a monophyly among Hippoboscoidea members and that the ancestor of this superfamily was a free-living insect feeding on mammal blood (Nirmala et al. 2001; Dittmar et al. 2006; Petersen et al. 2007).

Worldwide, more than 213 hippoboscid species are known (e.g., Maa 1963; Dick 2006; Rahola et al. 2011), and 31 species of Hippoboscidae have been described from Europe (Pape et al. 2015; Nartshuk et al. 2019a; Oboňa et al. 2019b).

**Figure 1.**
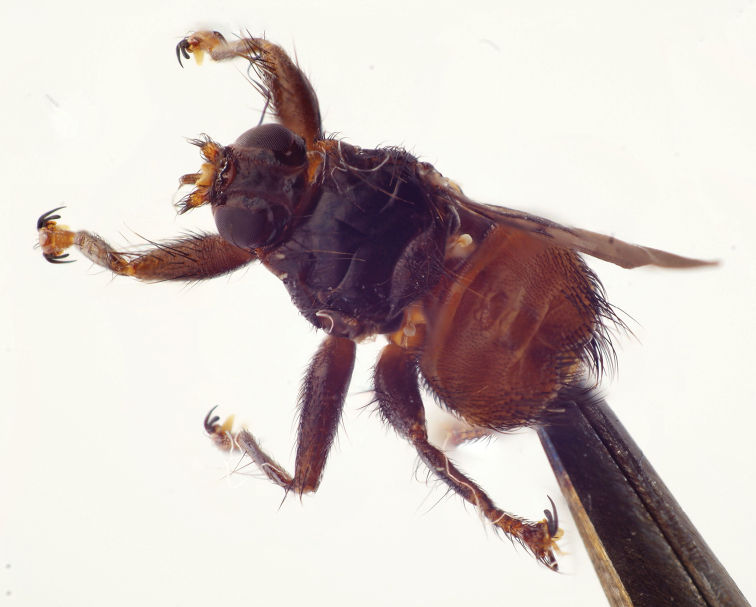
*Ornithomyacomosa*, imago, dorsal view (left wing removed).

**Figure 2.**
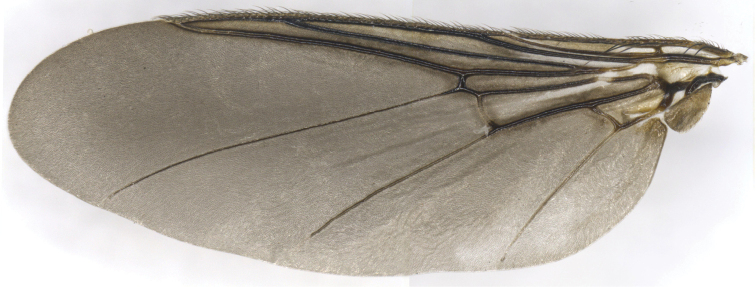
*Ornithomyacomosa*, wing.

*Ornithomyacomosa* (Austen, 1930) (Figs 1, 2), the most recent species found in Europe (see Nartshuk et al. 2019a) and originally described from India (Pusa, Bihar), was first collected from a grey-throated martin, *Ripariachinensis* (J. E. Gray, 1830) (Austen 1930). The host overview is presented in Table 1. According to Maa (1969a, b, 1977), *O.comosa* is distributed in India, Nepal (on *R.chinensis*; Maa (1969a) used the name *Ripariapaludicolachinensis*), and Thailand and Malaysia (on a barn swallow, *Hirundorustica* Linnaeus, 1758).

**Table 1.** The overview of hosts of *Ornithomyacomosa* (Austen, 1930).

Host species	Countries	References
*Cecropisdaurica*	Russia	Nartshuk et al. (2019b)
*Delichonurbicum*	Kazakhstan, Kyrgyzstan, Russia	Doszhanov (1970); Nartshuk et al. (2019a)
*Hirundorustica*	Japan, Kazakhstan, Kyrgyzstan, Malaysia, Russia, Thailand,	Maa (1969a); Doszhanov (1970); Mogi (2014); Nartshuk et al. (2019a)
*Otusscops*	Russia	Doszhanov (2003)
*Ripariadiluta*	Russia	Matyukhin and Gashkov (2020)
*Ripariachinensis*	India, Nepal	Austen (1930); Maa (1969a)
*Ripariariparia*	Japan, Kazakhstan, Kyrgyzstan, Russia	Doszhanov (1970); Mogi (2014)

Subsequently, Doszhanov (1970, 2003) recorded this species from Kazakhstan, Kyrgyzstan and West Siberia of Russia (Novosibirsk), mostly from the host *Ripariariparia* (Linnaeus, 1758), rarely from *H.rustica* and *Delichonurbicum* (Linnaeus, 1758), and also from the Eurasian scops owl, *Otusscops* (Linnaeus, 1758) of the Strigiformes. Mogi (2014) reported this species from Japan (Honshu, Kyushu and Ryukyu islands on *R.riparia* and *H.rustica* hosts). Most recently, Nartshuk et al. (2019a) recorded *O.comosa* for the first time in Europe from western Russia (Kaliningrad Province, hosts *H.rustica* and *D.urbicum*). Further Russian records are from *Cecropisdaurica* (Laxmann, 1769) from Primorskii krai in the Far East (Nartshuk et al. 2019b) and from *Ripariadiluta* (Sharpe & Wyatt, 1893) from Tomsk in west Siberia (Matyukhin and Gashkov 2020). In this study *O.comosa* is recorded for the first time from Slovakia, demonstrating its further expansion in Central Europe.

A series of new records of louse flies and keds from Slovakia with several new host records is appended to supplement the recent review by Oboňa et al. (2019b). Because the available taxonomic keys for European Hippoboscidae are outdated, we present updated keys covering all species currently known from Europe.

## ﻿Materials and methods

The key for European genera of Hippoboscidae follows the previous descriptions by Bequaert (1954), Theodor and Oldroyd (1964), and Hutson (1984). Keys for species of European Hippoboscidae follow Falcoz (1926), Povolný and Rosický (1955), Theodor and Oldroyd (1964), Maa (1966, 1969c), Hutson (1981, 1984), Ducháč and Bádr (1998), Farafonova (2001), Petersen et al. (2007), Iwasa and Choi (2013), Nartshuk et al. (2019b), and Salvetti et al. (2020).

In addition, new louse fly specimens from Slovakia were collected by hand on birds caught in mist nets, or keds by hand from humans. The majority of the samples come from the ornithological station “Vtáčí raj – Šalgovské rybníky” (Bird’s Paradise – Šalgov ponds) near the village Uzovský Šalgov (49°05'34.8"N, 21°04'00.4"E, 366 m a.s.l.). The birds were mist-netted in the standardised method (for more information, see Olekšák et al. 2007). Other samples, especially from humans, represent random and non-targeted sampling.

The collected hippoboscids were placed in microvials with 96% ethanol and subsequently identified in the laboratory using determination keys by Povolný and Rosický (1955) and Theodor and Oldroyd (1964). The focus on the local primary hosts follows Oboňa et al. (2019a, b, 2021). The newly recorded species *O.comosa* (Austen, 1930) was identified by Nartshuk et al. (2019b) using a key modified according Farafonova (2001). The material is deposited in the collection of the Laboratory and Museum of Evolutionary Ecology, Department of Ecology, University of Prešov (**LMEE PO**). The terminology follows Cumming and Wood (2017).

## ﻿Results

### ﻿Key for European genera of Hippoboscidae (updated)

**Table:** 

1	Wings fully developed and functional (Figs 3–11)	**2**
–	Wings reduced, with strong veins (Figs 12–16) or absent (either by reduction or loss)	**9**
2	Tarsal claws simple (Fig. 17), but with a pale basal lobe; humeral callus weak (Figs 21–24), postpronotum rounded, not produced anteriorly as conical lobes	**3**
–	Tarsal claw bifid and with a pale basal lobe (Fig. 18); humeral callus strong, postpronotum rounded, pair of conical lobes on either side of head (Figs 26–28), on birds	**5**
3	Wing with one or two cross-veins; R_4+5_ well separated from C until apex; on mammals (Figs 4, 5)	**4**
–	Wings with three cross-veins enclosing cells posterior to radial veins; apical 1/2 of vein R_4+5_ running very close to C (Fig. 3); on birds	***Ornithoica***
4	Wing clear and hyaline, with only one cross-vein (Fig. 4); head broader than long; thorax markedly flattened (Figs 21–25); on mammals	***Lipoptena***
–	Wing distinctly crenulated and tinted, with two cross-veins (Fig. 5); head not broader than long; thorax not so markedly flattened; on mammals	***Hippobosca***
5	Wing with three cross-veins posterior to radial veins (Figs 6, 7); scutellum with four or more strong marginal setae (Figs 32–37)	**6**
–	Wing with one or two cross-veins posterior to radius (Figs 8–11); scutellum at most with two strong marginal setae (Figs 31, 38)	**7**
6	Vein R_2+3_ with apical 3/5 fused with C; wing membrane entirely bare (Fig. 6)	***Ornithophila***
–	Vein R_2+3_ well separated from C except at apex; wing membrane usually with microtrichia (Fig. 7)	***Ornithomya***
7	Wing with only one cross-vein (Fig. 8)	***Pseudolynchia***
–	Wing with two cross-veins (Figs 9–11)	**8**
8	Scutellum with two strong setae (Fig. 19)	***Icosta***
–	Scutellum with setulae (Fig. 31)	***Olfersia***
9	Wing long and narrow, at least 6 × as long as wide and twice as long as head and thorax (Fig. 12); female abdomen with strong spiniform setae in posterolateral area; male abdomen without spiniform setae	***Stenepteryx***
–	Wing short and broad, at most 3 × as long as wide and ~ 1.5 × as long as head and thorax (Figs 13–16); tip of wing usually attenuated, C reaching to about 0.75 length of anterior wing margin; female abdomen only with short fine setae in posterolateral area	***Crataerina***
10	Wings either reduced to a veinless knob or broken off; haltere absent	***Melophagus***
–	Wings absent, leaving a broad flat veined stump; haltere present	***Lipoptena***

### ﻿Keys to species of European genera of Hippoboscidae (updated)

**﻿The genus *Crataerina* von Olfers, 1816**

**Table:** 

1	Wing shorter than hind femur; wing tip broadly rounded (Fig. 13)	***Crataerinaobtusipennis* Austen, 1926**
–	Wing longer than hind femur	**2**
2	Wing more than twice as long as hind femur (Fig. 14); male abdomen with tergites 3 and 4 one-third as wide as abdomen and tergite 5 nearly as wide as abdomen; female abdomen with long and thick setae on posterior margin and with group of fine and long setae ventral to genital opening	***Crataerinamelbae* (Rondani, 1879)**
–	Wing < 2 × as long as hind femur; male abdominal tergites small or absent; all setae on posterior margin of female abdomen short and uniform in length	**3**
3	Wing length 2 × as long as hind femur, extended beyond posterior end of abdomen; distal 1/2 of trailing edge of wing strongly concave (Fig. 15)	***Crataerinaacutipennis* Austen, 1926**
–	Wing length 1.3–1.5 × as long as hind femur, not extended beyond posterior end of abdomen; distal 1/2 of trailing edge of wing not strongly concave (Fig. 16)	***Crataerinapallida* (Olivier in Latreille, 1811)**

Host-parasite associations: Aves (Apodiformes, Passeriformes).

### ﻿The genus *Hippobosca* Linnaeus, 1758

**Table:** 

1	Vein R_2+3_ meets vein C at same place as R_1_, shorter than distal section of R_4+5_ (measured from transverse vein r-m); front edge of thorax, with a transverse row of short thick setae; scutellum almost rectangular, with 2 dark and 3 light spots; wing length 7.0–8.0 mm	***Hippoboscavariegata* Megerle, 1803**
–	Vein R_2+3_ end into vein C clearly separated from R_1_, length is approximately equal to distal section of vein R_4+5_; thorax without mentioned setae and characters; wing length shorter than 7.0–8.0 mm	**2**
2	Dark brown specimens; veins of wings dark pigmented; scutellum white in middle, dark on sides; wing length 6.0–8.5 mm (Fig. 5)	***Hippoboscaequina* Linnaeus, 1758**
–	Pale specimens; veins of wings light, only transverse veins and sections of longitudinal veins adjoining them are completely or partially dark; scutellum almost entirely white, sometimes with dark edge; wing length 5.0–6.0 mm	***Hippoboscalongipennis* Fabricius, 1805**

Host-parasite associations: Aves (Accipitriformes), Mammalia (Carnivora, Cetartiodactyla, Perissodactyla).

### ﻿The genus *Icosta* Speiser, 1905

**Table:** 

1	Large dark specimens; wing length 5.0–6.0 mm	**2**
–	Small pale specimens; wing length 3.5–4.0 mm	**3**
2	Venter of hind femur bare; palp length more than twice width; microtrichia covering most of wing, but apical 1/2 of cell Cu+1A and entire 2A bare (Fig. 10); prescutum with setae reaching mesonotal suture; pale yellowish specimens; abdomen without tergite 3 (Fig. 39)	***Icostaminor* (Bigot in Thomson, 1858)**
3	Venter of hind femur densely setose except near base; length of palp ~ 1.5 × width; wing with microtrichia covering most of its surface, including anterior 1/3 of cell 2A (Fig. 9); prescutum with short setae in several rows not reaching mesonotal suture, smaller and disordered short postalar setae in several rows (Fig. 19); dark specimens; abdomen with distinct tergite 3 (Fig. 40)	***Icostaardeae* (Macquart, 1835)**
–	Enigmatic species, so far known from a single specimen; prescutum with one row of fine longer setae that reach mesonotal suture, setae in one row (Fig. 20)	***Icostamassonati* (Falcoz, 1926)**

Host-parasite associations: Aves (Passeriformes, Pelecaniformes).

### ﻿The genus *Lipoptena* Nitsch, 1818

**Table:** 

1	Wing length 6.0 mm	**2**
–	Wing length 4.0 mm or less	**3**
2	Body length 5.0–6.0 mm; scutellum with 6–8 setae; thorax mostly with 30–35 setae on each side, 9 postalar setae on each side (Fig. 21)	***Lipoptenacervi* (Linnaeus, 1758)**
–	Body length 4.5–5.5 mm; scutellum with 8–10 setae; thorax mostly with 50–60 setae on each side, 6 postalar setae on each side (Fig. 22)	***Lipoptenacouturieri* Séguy, 1935**
3	Wing length 4.0 mm; body length 2.8–3.2 mm; scutellum with 4–6 setae; thorax mostly with 8–12 strong setae on each side, 4 postalar setae on each side (Fig. 23)	***Lipoptenafortisetosa* Maa, 1965**
–	Wing length < 4.0 mm; scutellum with 6 setae; thorax mostly with 25 or more setae on each side, 3 or 4 postalar setae on each side	**4**
4	Wing length 3.0–3.2 mm; body length 3.0–3.75 mm; body pale; thorax mostly with 30–35 setae on each side (Fig. 24)	***Lipoptenacapreoli*Rondani, 1878**
–	Wing length < 3.0 mm body length 2.3–2.6 mm; body extremely dark; thorax mostly with 25–30 soft setae on each side (Fig. 25)	***Lipoptenaarianae* Maa, 1969**

Host-parasite associations: Mammalia (Cetartiodactyla, Carnivora).

### ﻿The genus *Melophagus* Latreille, 1802

**Table:** 

1	Palps almost as long as head, in rest position completely covering proboscis (Fig. 29); parafrontalia almost touching in middle, mediovertex reduced; parafrontalia covered with numerous setae; tergal plates completely absent in male, in females only remnants of tergal plate 7	***Melophagusovinus* (Linnaeus, 1758)**
–	Palps shorter than head, ~ 1/3 of head length, proboscis always protruding (Fig. 30); parafrontalia with few setae on inner margin; in males tergal plate 6 present, in females plates 6 and 7 present	***Melophagusrupicaprinus*Rondani, 1879**

Host-parasite associations: Mammalia (Carnivora, Cetartiodactyla, Perissodactyla).

**Figures 3–16.**
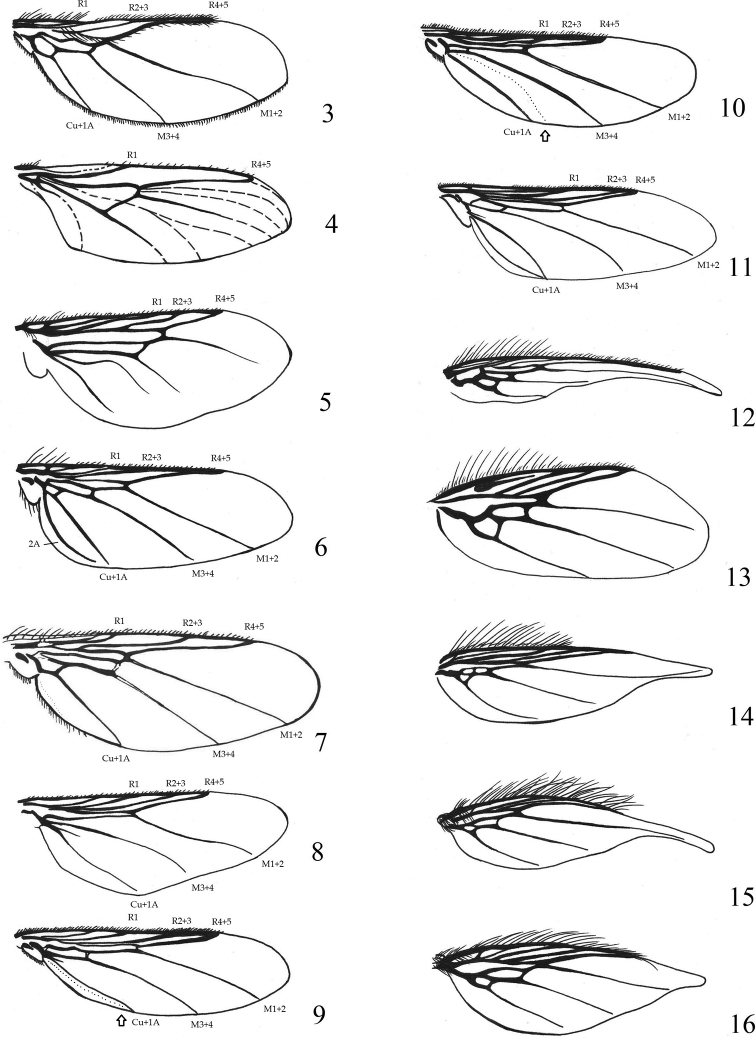
**3***Ornithoicaturdi*, wing **4***Lipoptenacervi*, wing **5***Hippoboscaequina*, wing **6***Ornithophilametallica*, wing **7***Ornithomyaavicularia*, wing **8***Pseudolynchiacanariensis*, wing **9***Icostaardeae*, wing (with the border of microtrichia) **10***Icostaminor*, wing (with the border of microtrichia) **11***Olfersiaspinifera*, wing **12** S*tenepteryx hirundinis*, wing **13***Crataerinaobtusipennis*, wing **14***Crataerinamelbae*, wing **15***Crataerinaacutipennis*, wing **16***Crataerinapallida*, wing.

**Figures 17–24.**
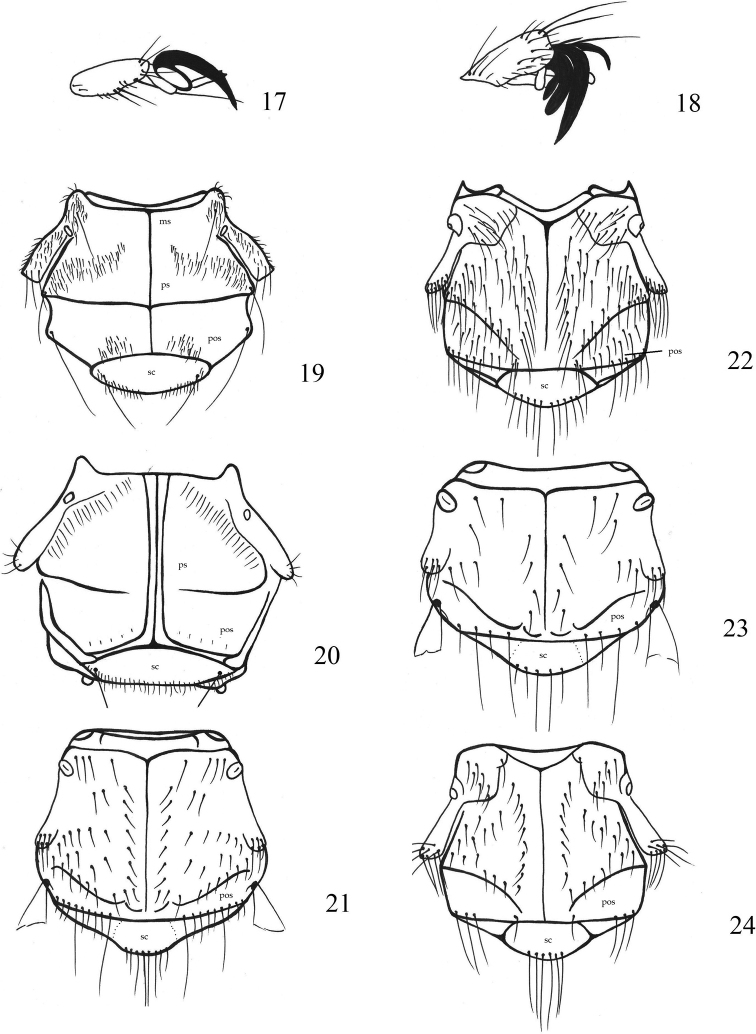
**17***Lipoptenacervi*, tarsal claws **18***Ornithomyaavicularia*, tarsal claws **19***Icostaardeae*, thorax **20***Icostamassonati*, thorax **21***Lipoptenacervi*, thorax **22***Lipoptenacouturieri*, thorax **23***Lipoptenafortisetosa*, thorax **24***Lipoptenacapreoli*, thorax. Abbreviations: ms – mesonotal suture, pos – postalar setae, ps – prescutum, sc – scutellum.

**Figure 25–30.**
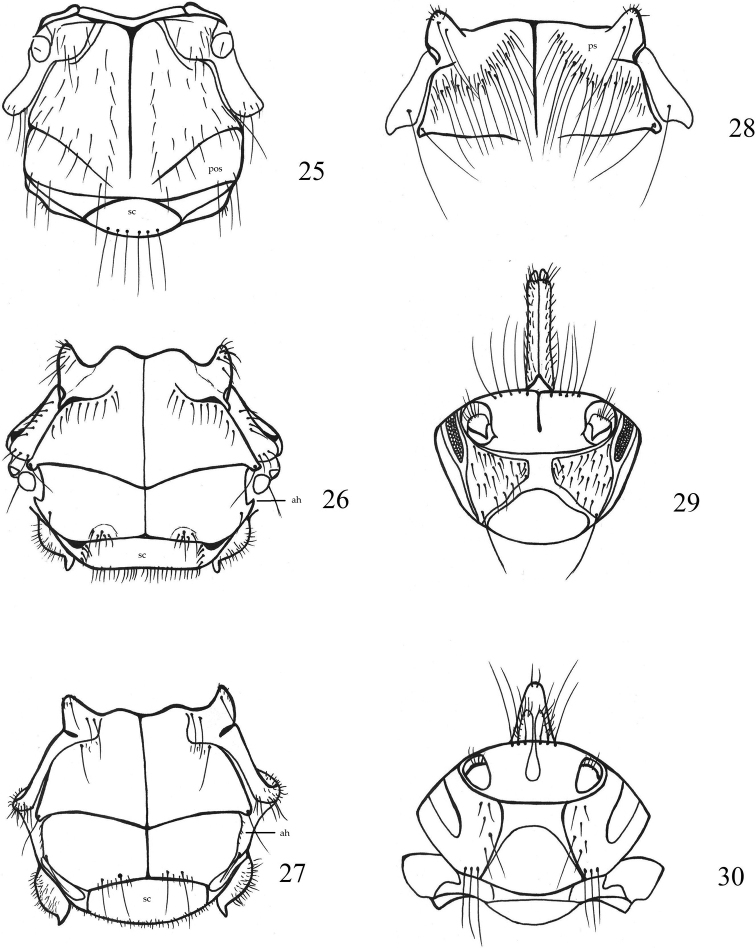
**25***Lipoptenaarianae*, thorax **26***Olfersiaspinifera*, thorax **27***Olfersiafumipennis*, thorax **28***Pseudolynchiacanariensis*, frontal part of thorax **29***Melophagusovinus*, head with palps **30***Melophagusrupicaprinus*, head with palps. Abbreviations: ah – alar horns, pos – postalar setae, ps – prescutum, sc – scutellum.

### ﻿The genus *Olfersia* Wiedemann, 1830

**Table:** 

1	Head on posterior margin with 3 distinct protrusions and deep indentations between postvertex and posterior orbit; postvertex protrudes noticeably backwards over posterior orbites; tip of alar horns directed obliquely forward (Fig. 26); section of C between Sc and R_1_ is shorter than section between R_1_ and R_2-3_. First basal cell is ca. as long as section of R_4+5_ distal to transverse vein r-m (Fig. 11); female pygidium separate and finger-shaped	***Olfersiaspinifera* (Leach, 1817)**
–	Head protruding only a little over posterior orbit; tip of alar horns blunt and serrated (Fig. 27); sections of C between Sc and R_1_ longer than section between R_1_ and R_2+3._ First basal cell is significantly shorter than section of R_4+5_ distal to transverse vein r-m; female pygidium short and fused	***Olfersiafumipennis* (Sahlberg, 1886)**

Host-parasite associations: Aves (Accipitriformes, Gaviiformes, Charadriiformes, Pelecaniformes, Suliformes).

### ﻿The genus Ornithoica*Rondani*, 1878 – one species only

***Ornithoicaturdi* (Olivier in Latreille, 1812)**

Figs 3, 32

Host-parasite associations: Aves (Passeriformes).

### ﻿The genus *Ornithomya* Latreille, 1802

**Table:** 

1	C sector between R_1_ and R_2+3_ not longer than sector between R_2+3_ and R_4+5_	**2**
–	C sector between R_1_ and R_2+3_ longer than between R_2+3_ and R_4+5_	**3**
2	Brown spots on ventral side of head do not reach jugular setae; scutellum with 4 setae (Fig. 33); wing in hind part with 4 longitudinal stripes of microtrichia; adult 1.9–2.5 mm	***Ornithomyafringillina* Curtis, 1836**
–	Triangular brown spots on ventral side of head are sharp (Fig. 41), narrowed and reach jugular setae, which are situated on sides of occipital foramen; scutellum usually with 6 or more setae (Fig. 34); wing in hind part with 3 longitudinal stripes of microtrichia; adult 2.1–2.6 mm	***Ornithomyachloropus* Bergroth, 1901**
3	Wing dark and all surface evenly covered by microtrichia; scutellum with 10–12 setae (Fig. 35); all body covered by setae; adult 2.0–2.5 mm	***Ornithomyacomosa* (Austen, 1930)**
–	Surface of wing covered by microtrichia no more than 2/3, base of wing without of microtrichia	**4**
4	Wing with microtrichia only on apex and in cell m_1_; scutellum with 8 setae (Fig. 36); abdomen on apex with numerous long setae; adult 3.0–3.5 mm	***Ornithomyaavicularia* (Linnaeus, 1758)**
–	Microtrichia covered nearly all wing except base or only cells r_3_ and m_2,_ long setae absent on apex of abdomen	**5**
5	Wing dark with intensive microtrichia; thorax with 16–18 mesopleural setae on each side; scutellum with 6 setae (Fig. 37); abdomen similar as in following species; vibrissal spines almost missing	***Ornithomyabiloba* Dufour, 1827**
–	Wing light with extensive microtrichia, thorax with 6–10 mesopleural setae on each side; scutellum with 4 (6) setae; abdomen (Figs 43, 44); vibrissal spines present (Fig. 42)	***Ornithomyarupes* Hutson, 1981**

Host-parasite associations: Aves (Accipitriformes, Anseriformes, Falconiformes, Passeriformes, Pelecaniformes, Strigiformes), Mammalia (Primates).

### ﻿The genus *Ornithophila*Rondani, 1879

**Table:** 

1	4.0–5.0 mm; scutellum dark, except for a narrow, light stripe at base; male tergal plates 3 and 4 as wide as scutellum	***Ornithophilametallica* (Schiner, 1864)**
–	5.0–7.0 mm; scutellum with a broad yellow band at base and a yellow triangle at apex; male tergal plates 3 and 4 as wide as a little more than 1/2 width of scutellum	***Ornithophilagestroi* (Rondani, 1878)**

Host-parasite associations: Aves (Passeriformes).

### ﻿The genus *Pseudolynchia* Bequaert, 1926

**Table:** 

1	Hind scutellar margin in dorsal view straight or nearly straight (Fig. 38); interantennal area of frons as wide as or rarely slightly narrower than its distance to eye; prescutum with 20–30 long pale fine setae and before which with 2 or 3 series of shorter ones (Fig. 28); mid tarsus with group of peg-like modified spines under segment 1 at base	***Pseudolynchiacanariensis* (Macquart in Webb & Berthelot, 1839)**
–	Hind scutellar margin in dorsal view distinctly curved; interantennal area of frons always much narrower than its distance to eye; prescutum with 12–18 long, fairly robust and generally black setae and before which, with 1 or 2 series of shorter ones; mid tarsus with only pointed setae under segment 1 at base	***Pseudolynchiagarzettae* (Rondani, 1879)**

Host-parasite associations: Aves (Accipitriformes).

### ﻿The genus *Stenepteryx* Leach, 1817 – one species only

***Stenepteryxhirundinis* (Linnaeus, 1758)**

Fig. 12

Host-parasite associations: Aves (Passeriformes: Hirundinidae).

**Figures 31–38.**
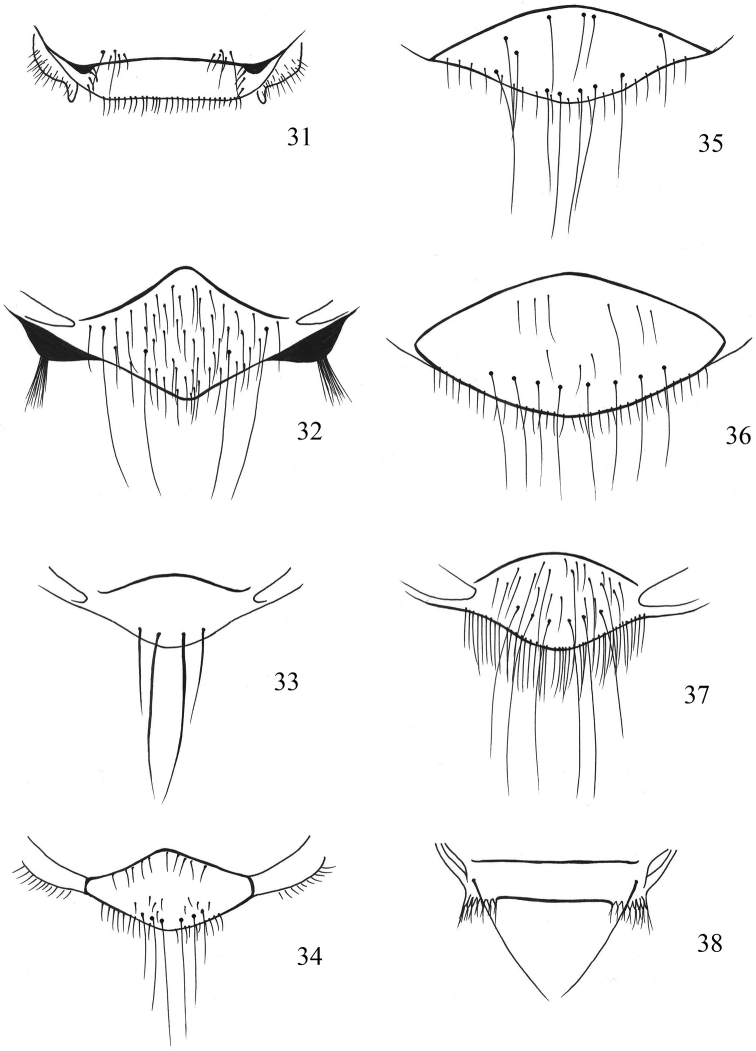
**31***Olfersiafumipennis*, scutellum **32***Ornithoicaturdi*, scutellum **33***Ornithomyafringillina*, scutellum **34***Ornithomyachloropus*, scutellum **35***Ornithomyacomosa*, scutellum **36***Ornithomyaavicularia*, scutellum **37***Ornithomyabiloba*, scutellum **38***Pseudolynchiacanariensis*, scutellum.

**Figures 39–44.**
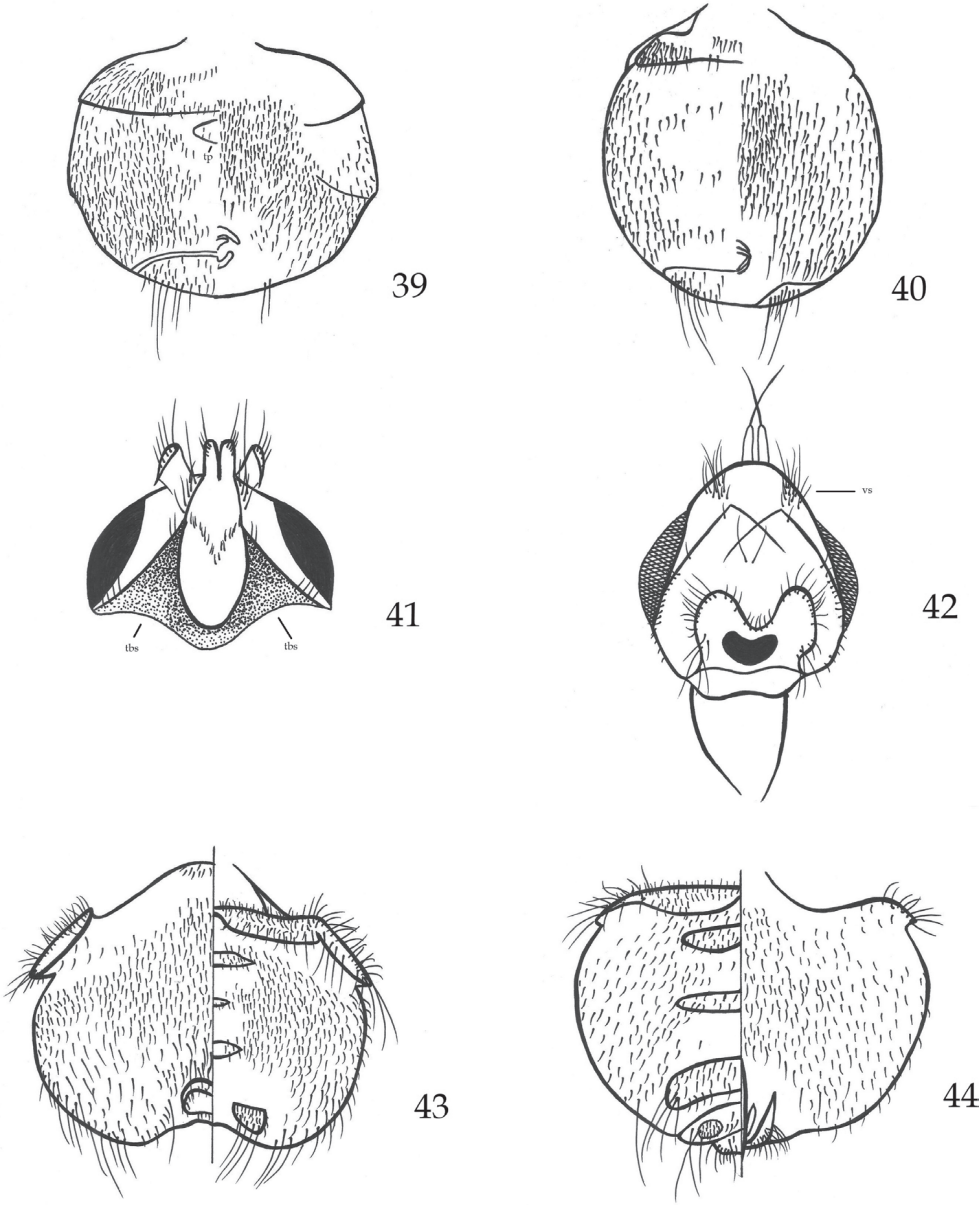
**39***Icostaminor*, ♀ abdomen, dorsal and ventral view **40***Icostaardeae*, ♀ abdomen, dorsal and ventral view **41***Ornithomyachloropus*, head, ventral view **42***Ornithomyarupes*, head, ventral view **43***Ornithomyarupes*, ♀ abdomen, dorsal and ventral view **44***Ornithomyarupes*, ♂ abdomen, dorsal and ventral view. Abbreviations: tbs – triangle brown spot, tp – tergal plate, vs – vibrissal spines.

## ﻿Discussion

Updated keys to eleven European hippoboscid genera comprising 31 species are provided. We hope that they will contribute as a tool for determining European specimens of the most fascinating and neglected group of blood-feeding ectoparasites from the family Hippoboscidae (see Bezerra-Santos and Otranto 2020).

The recent checklist of louse flies of the family Hippoboscidae from Slovakia (see Oboňa et al. 2019b) includes 19 species; the present paper increases this list to 20 species. Four new host-parasite associations from Slovakia are also recorded (*O.avicularia* on the reed bunting and European starling, and *O.fringillina* on the barn swallow and the lesser whitethroat; see also Table 2).

**Table 2.** An overview of recorded host-parasites associations.

Parasites	Hosts
*Hippoboscaequina*	Mammalia: Primates: Homosapiens*
*Lipoptenacervi*	Mammalia: Primates: Homosapiens*
*Lipoptenafortisetosa*	Mammalia: Primates: Homosapiens*; Aves: Parusmajor*
*Ornithomyaavicularia*	Aves: Passeriformes: *Emberizaschoeniclus*, *Hirundorustica*, *Panurusbiarmicus*, *Sturnusvulgaris*
*Ornithomyabiloba*	Aves: Passeriformes: *Hirundorustica*, *Ripariariparia*
*Ornithomyacomosa*	Aves: Passeriformes: *Ripariariparia*
*Ornithomyafringillina*	Aves: Passeriformes: *Currucacurruca*, *Hirundorustica*
*Stenepteryxhirundinis*	Aves: Passeriformes: *Delichonurbicum*

* Accidental association, parasite species do not complete development on this host.

Of a total of 20 Slovakian hippoboscid species, 12 are native. The remaining eight species (*Hippoboscalongipennis* Fabricius, 1805, *H.variegata* Megerle, 1803, *Icostaminor* (Bigot, 1858), *Olfersiafumipennis* (Sahlberg, 1886), *Ornithoicaturdi* (Latreille, 1812), *Ornithophilametallica* (Schiner, 1864), *Pseudolynchiacanariensis* (Macquart, 1839), and the newly recorded *Ornithomyiacomosa* (Austen, 1930) have been recorded from Slovakia based on very few records due to their hosts being usually occasional visitors (Oboňa et al. 2019b).

According to Nartshuk et al. (2019a), there are two possible explanations for the current distribution of *O.comosa*: *O.comosa* migrates with adult swallows from West Siberia or Kazakhstan to western Russia or *O.comosa* has always or for a long time been present in western Russia, but it has not been previously collected. For the European records of *O.comosa*, migrating swallows cannot bring *O.comosa* from Africa, as *O.comosa* does not occur there, but some *H.rustica* do, spending the winter in Asia, where *O.comosa* occurs. We also assume that records by Doszhanov (1970, 2003) from Kazakhstan and Kyrgyzstan could represent a bridge between the East Asian-Australasian Flyway and the African-Eurasian Flyway (see Boere and Stroud 2006). In this area, parasites can be transferred between hosts from East Asian countries (e.g., India, Nepal, Thailand, Malaya, Japan) to western Siberia, western Russia, and Europe. However, the question is whether *O.comosa* has already adapted to local conditions and become a native species in Europe or will it continue to be only an occasionally introduced species.

The collection dates by Austen (1930) from India (February–April), King (1969) from Thailand (Bangkok) (ectoparasites collected during “winter”), and Mogi et al. (2002) from Japan (September–December) suggest that *O.comosa* is active mainly in autumn and winter (native populations). However, the findings from Kazakhstan by Doszhanov (1970) are from the period May–October, and the most recent records from the Kaliningrad region (Nartshuk et al. 2019a) correspond to the findings presented in this article, i.e., July and August. Therefore, we believe that the parasite does not have to come from the spring migration but, on the contrary, from the summer/autumn migration (possibly also from the Kaliningrad area). A similar phenomenon is recorded for the non-native species *Ornithoicaturdi* (Olivier in Latreille, 1811) (e.g., records from Vienna from August, see Zittra et al. (2020)) transported by hosts from wintering grounds in Africa. The relatively late record after migration from the wintering grounds has two possible explanations. The first is that these parasites are able to live for more than two months (see Chalupský 1980). The second is that it could be a hatched fly, and therefore it may not be a casual traveller from Africa (*O.turdi*) or Asia (*O.comosa*), but that it is already breeding here (as a native species?). This is also indirectly confirmed by the negative findings from the spring migration.
